# Synthesis and Characterization of Novel Sprayed Ag-Doped Quaternary Cu_2_MgSnS_4_ Thin Film for Antibacterial Application

**DOI:** 10.3390/nano12193459

**Published:** 2022-10-03

**Authors:** Amal Hammoud, Mehdi Souli, Mohamed Fethi Diouani, Badriyah Alhalaili, Ruxandra Vidu, Najoua Kamoun-Turki

**Affiliations:** 1 LR99ES13, Laboratoire de Physique de la Matière Condensée (LPMC), Département de Physique, Faculté des Sciences de Tunis, Université Tunis El Manar, Tunis 2092, Tunisia; 2 LR16IPT03, Laboratory of Epidemiology and Veterinary Microbiology (LEMV), Institut Pasteur de Tunis, Tunis-Belvédère 1002, Tunisia; 3 Nanotechnology and Advanced Materials Program, Kuwait Institute for Scientific Research, P.O. Box 24885, Safat 13109, Kuwait; 4 Faculty of Materials Science and Engineering, University POLITEHNICA of Bucharest, 060042 Bucharest, Romania; 5 Department of Electrical and Computer Engineering, University of California Davis, Davis, CA 95616, USA

**Keywords:** Ag doping, Ag incorporation, antibacterial activity, Cu_2_MgSnS_4_, impedance spectroscopy, spray pyrolysis, structural properties

## Abstract

In this work, the effects of silver doping with different Ag/(Ag + Cu) ratios (i.e., 2%, 5% and 10% at.% in the spray solution) on the structural, morphological, optical, electrical and antibacterial properties of Cu_2_MgSnS_4_ (CMTS) thin film grown by spray pyrolysis have been studied. The X-ray diffraction (XRD) and selected area electron diffraction (SAED) results have shown that the kesterite phase of CMTS thin films has a maximum crystallite size of about 19.60 nm for 5% Ag/(Ag + Cu). Scanning electron microscopy (SEM) images have shown spherical grain shapes. The transmission electron microscopy (TEM) and high-resolution TEM (HRTEM) microscopy observations confirmed the intrinsic reticular planes of CMTS thin film with (112) as a preferred orientation and interplanar spacing value of 3.1 Å. The optical properties showed high absorbance and an absorption coefficient of about 10^4^ cm^−1^ in the visible region with an optical band gap energy of 1.51 eV. Impedance analysis spectroscopy demonstrated good electrical properties of the CMTS film obtained using 5% Ag/(Ag + Cu). The antibacterial activity of the undoped and Ag-doped particles of CMTS obtained using 5% Ag/(Ag + Cu) against different strains of pathogenic bacteria was tested using the agar well diffusion method. These results showed a significant antibacterial activity of the Ag-doped CMTS particle, which was much higher than the undoped CMTS particles. These experimental findings may open new practices for the Ag-doped CMTS compound, especially the one obtained using 5% Ag/(Ag + Cu), in antibacterial application.

## 1. Introduction

Environmentally friendly, earth-abundant elements and non-toxic compounds are some of the key advanced materials that enable a sustainable future. Quaternary chalcogenides such as Cu_2_MgSnS_4_ (CMTS) have attracted enormous attention in various fields including photovoltaic and photocatalytic [1,2] applications. The main advantages of this material are its high photostability [2], absorption coefficient greater than 10^4^ cm^−1^ [3] and a remarkable direct energy gap of 1.5–1.7 eV [1,3,4]. In fact, CMTS is a quaternary compound that has an interesting combination of earth-abundant and low-toxic elements.

CMTS thin films have been grown using various techniques, such as hot injection [4], ultrasonic co-spray [3], spin-coating with and without sulfurization [2,5], pulsed laser deposition [6] co-precipitation [1,7] and pulverization in liquid phase (spray pyrolysis) [8,9,10]. More recently, the spray pyrolysis technique has begun to be considered as a promising method due to its low cost and simplicity.

**Figure 1:**
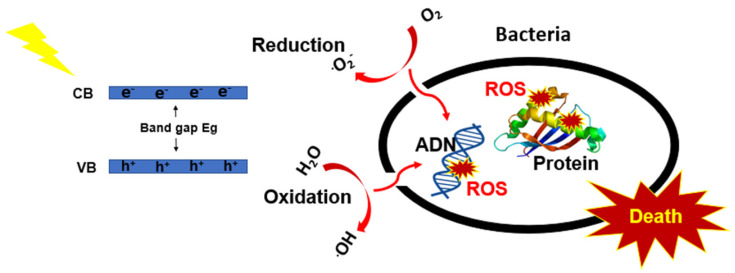
Photocatalytic mechanism for antibacterial activity.

Ali et al. [7] fabricated CMTS films with different Mg contents using co-precipitation method and demonstrated the high photocatalytic efficiency by degrading 70% of methylene blue (MB) and malachite green (MG) in 30 min under light irradiation [7]. In another work, Ali et al. [1] synthesized BaTiO_3_-decorated Cu_2_MgSnS_4_ as a potential material for removing organic contaminants from water, in addition to its resistance against pathogenic bacteria. Furthermore, the CMTS compound proves its use in water splitting applications as well as in photoelectrochemical cells due to its good photosensitivity as reported by Sharma et al. [2]. The research and development of antibiotic-resistant bacteria are rapidly developing to meet the demand for materials with low cost, high sensitivity and removal efficiency. Semiconductor (Sc) materials show great benefits over organic ones [11,12,13,14,15,16]; as shown in Figure 1, when Sc materials absorb light, the electron (e^−^) jumps from the valence band to conduction band leaving a hole (h^+^) behind, e^−^ can react with O_2_ to form superoxide radicals •O^−^_2_ and the holes react to generate hydroxyl radicals (•OH) [17]. Then, the reactive oxygen species (ROS) are created, which can inactivate the growth of bacteria or kill it [18].

Semiconductor oxides such as TiO_2_ and ZnO have noticeable antibacterial activity, especially when they are decorated with silver nanoparticles, as reported by Nguyen et al. [11]. Chalcogenide materials such as Cu_2_ZnSnS_4_ have been proved by Kumer et al. [19] to possess antibacterial properties. Additionally, Cu_2_MgSnS_4_ has already demonstrated its antibacterial activity [1]. CMTS is a semiconductor inorganic compound with a near infrared (NIR) band gap that can absorb the entire visible and NIR spectrum. The advantage of CMTS is that it contains nontoxic elements and its low band gap can lead to easy ROS generation and improved antimicrobial activities. 

**Figure 2:**
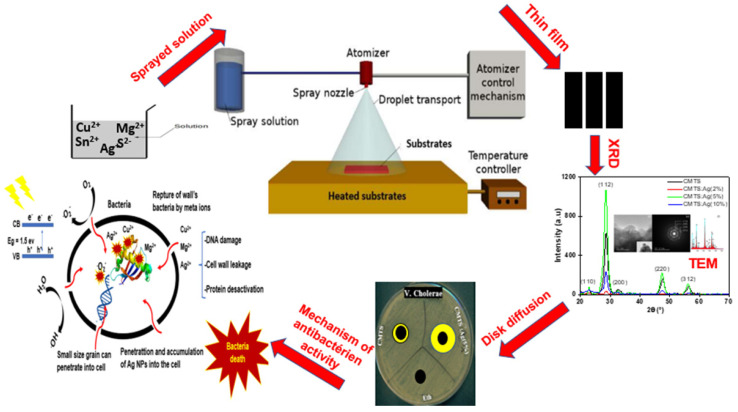
Rationale of the work performed on CMTS and Ag–doped CMTS thin films for antibacterial activity.

In this work, we grew CMTS films by the spray pyrolysis technique using an ethanolic solvent. To the best of our knowledge, there are no literature data regarding the Ag incorporation in the CMTS structure or its effects on the properties of the CMTS film. Therefore, we performed a comprehensive analysis of the structural, morphological, optical, electrochemical and electrical properties before and after doping for different Ag/(Ag + Cu) ratios (i.e., 2%, 5% and 10%) in the spray solution. This study was also carried out with the aim of finding out the role of Ag doping of CMTS on the antibacterial activity (Figure 2). For this purpose, CMTS and Ag-doped CMTS were tested on eight types of bacteria using the agar well diffusion method by determining the zone of inhibition (ZOI).

## 2. Materials and Methods

### 2.1. Preparation of CMTS Films

**Figure 3:**
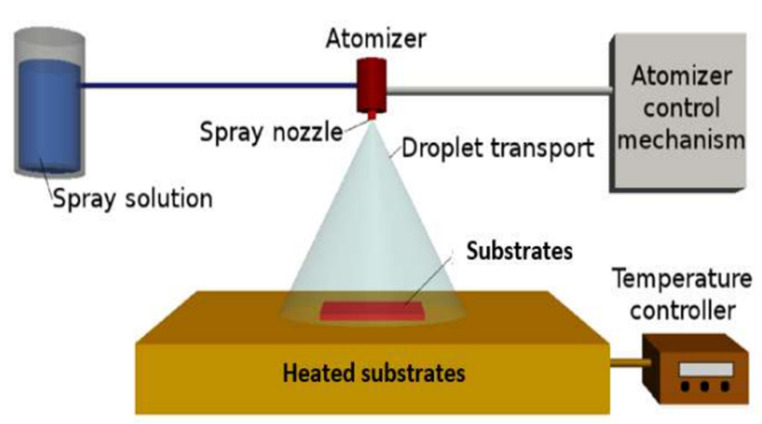
Schematic of the spray pyrolysis technique.

CMTS thin films have been deposited on glass substrates by the spray pyrolysis technique, as shown in Figure 3. Before the deposition, the glass substrates were ultrasonically cleaned in bi-distilled water and dried at 60 °C. The spray solution rate was fixed to 20 mL/min. The spraying system is equipped with a spray nozzle [20], air pressure controller, precursor solution, liquid flow meter, substrate heater and temperature controller. An ethanolic solution of 300 mL was formed containing: 10^−2^ mol/L of copper (II) chloride dihydrate (CuCl_2_.2H_2_O) from AppliChem GmbH Ottoweg 4 D-64291 Darmstat (Darmstadt, Germany); 5.10^−3^ mol/L of magnesium (II) chloride hexahydrate (MgCl_2_, 6H_2_O, 99%) from AppliChem (Chicago, IL, USA); 5.10^−3^ mol/L of tin (II) chloride dihydrate (SnCl_2_.2H_2_O) from Riedel-de Haen, Sigma Aldrich Laborchemikalien GmbH (Wunstorfer, Germany); 40.10^−3^ mol/l of thiourea (CH_4_N_2_S, 97.0%) from Fulka Chemilka, Sigma Aldrich Chemie GmbH (St. Gallen, Switzerland) as precursors for Cu, Mg, Sn and S. To this solution, AgNO_3_ was added in various Ag/(Ag + Cu) ratios in the spray solution, i.e., 2%, 5% and 10% at.%. Secondly, the solution was aspirated using a pump and passed through a nozzle in which the liquid was broken into small droplets. The droplets were then transported and landed on the substrate, which was heated by a hot plate at 210 °C.

### 2.2. Film Characterization

CMTS structure was characterized by X-ray diffraction analysis (XRD), using XPERT-PRO Diffractometer system from 20° to 80°. The surface morphology modifications were investigated using a Quanta 200 scanning electron microscope (SEM) with 3 nm resolution at 30 kV and a Tecnai transmission electronic microscope (TEM) (G20, 200 kV). The optical data were identified using a Perkin Elmer Lambda 950 spectrophotometer in the range of 300–1800 nm. Finally, the electrochemical properties of CMTS films were studied by electrochemical impedance spectroscopy (EIS) using an Agilent E4980A impedance analyzer at room temperature in the frequency range of 20 Hz–2 MHz with a Xenon 100 W filament bulb as a light source.

### 2.3. Experimental Setup of Antibacterial Test

The agar-well diffusion method was used to evaluate the antibacterial activity towards eight strains of bacteria, such as *Vibrio cholerae CECT 8265* (V. Cholerae), *Enterococcus faecolis ATCC 11700* (*S. enterococcus*), *Shigella flexnerie ATCC 29903* (Shigella), *Pseudomonas aeruginosa ATCC 9027* (Pseudomonas), *Klebsiella oxytoca CECT 8207* (Klebsiella), *Staphylococcus aureus ATCC 6538* (Staph. Aureus), *Salmonella anatum ATCC 9270* (S. anatum), *Salmonella typhi ATCC14028* (S. Typhii), which were available as reference strains from the Laboratory of Epidemiology and Veterinary Microbiology at the Pasteur Institute of Tunis. In order to obtain a bacterial suspension, bacteria were first extracted using a wire loop and then placed in a sterile test tube. Then, bacteria were gently inoculated on the plate using a sterile cotton swab to create a homogenous layer of bacteria. 

In one plate, we put three filter paper discs of 8 mm diameter, which were loaded with 50 µL of solution containing 15 mg/mL of Ag-doped CMTS obtained using 5% Ag/(Ag + Cu) and ethanol as negative control. To obtain the CMTS particle dispersion, the CMTS and Ag-doped CMTS films were scratched. Then, the plates were kept in incubation at 37 °C for 24 h under dark or under light irradiation using two LED lamps. During the incubation process, bacteria grow across the plate except for the area around the paper disks, which is called the inhibition zone, where the concentration is high enough to inhibit their growth. After incubation, the antibacterial activities of the CMTS particles were evaluated by measuring the diameter of the inhibition around the discs, i.e., the inhibition zone. 

## 3. Results and Discussion

### 3.1. Structural Analysis

#### 3.1.1. XRD Analysis

**Figure 4:**
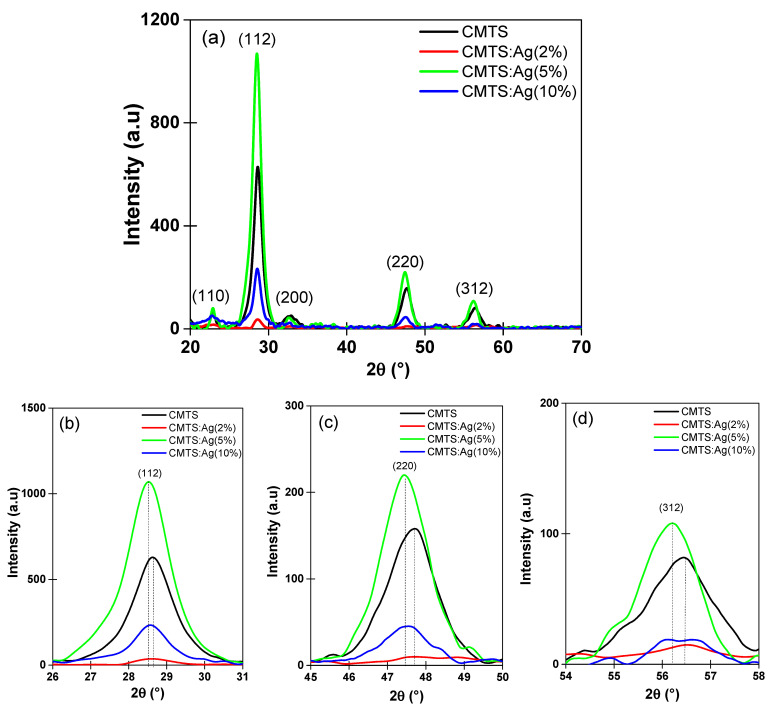
XRD patterns of the undoped and Ag-doped CMTS films obtained with 2%, 5% and 10% Ag in the spray solution (**a**). The magnified images of the peaks at 2θ = 28.5°, 47.4° and 56.5° are shown in (**b**–**d**), respectively.

Figure 4a shows the XRD patterns of undoped and Ag-doped CMTS thin films with different concentrations of Ag (2%, 5%, and 10%). In Figure 4a, different peaks located at 22.8°, 28.5°, 32.6°, 47.4° and 56.3° assigned to the reticular plans of (110), (112), (200), (220) and (312), respectively, which can be related to the Kesterite phase structure according to PDF 98–017–1983 of the International Center of Diffraction Data (ICDD) [1,2,9,10].

A noteworthy decrease in the intensity of the XRD diffraction of the preferred orientation (112) was observed when 2% Ag was added to the CMTS spray solution (Figure 1 and Figure 4a). Then, a substantial increase in the intensity of (112) peak was observed for 5% Ag, which was much higher than the undoped CMTS, followed by a drop in peak intensity when the Ag concentration in the solution reached 10% (Figure 4a,b). The same behavior has been observed for the other principal reticular plans (220) and (312) (Figure 4c,d). The XRD results show that adding 5% Ag to the spray solution improves the structural properties of CMTS. 

These results confirm that adding 5% Ag in the CMTS solution helps to improve the crystalline structure without introducing secondary phases, as reported in previous works on Ag-doped copper zinc tin sulfide CZTS [21,22], which belongs to the same family of quaternary compounds. Moreover, a small shift to the left was observed in Figure 4b–d for all of the diffraction angles for (112), (220) and (312), as the percentage of Ag in the CMTS films increases, which may be caused by (i) a possible rearrangement of atoms in the cell, (ii) distortions in the distance between reticular planes of the CMTS crystal lattice as mentioned by Chandel et al. [23], or (iii) local deformation of the crystal lattice due to the increase in Ag content, which has a larger ionic radius (1.14 Å) than that of Cu (0.74 Å) [22]. More precisely, Ag atoms may occupy various sites in the crystal lattice, in particular vacant sites, thereby improving the CMTS crystal structure at 5% Ag. On the other hand, an increase in silver content may force the Ag atoms to occupy interstitial sites, which causes the deterioration of CMTS crystallinity. Nguyen et al. [24] have reported similar results for Ag-containing CZTS.

The crystallite size (*D*), dislocation density (*δ*) and lattice strain (*ε*) were calculated using the following equations [9,20]:

(1) $$D=\frac{K\lambda }{\beta cos\left(\theta \right)}$$

(2) $$\delta =\frac{1}{D^{2}}$$

(3) $$\varepsilon =\frac{\beta cos\left(\theta \right)}{4}$$

where *λ* is the X-ray wavelength (1.5418 Å), *K* is the shape factor (*K* = 0.94), *β* is the full width at half maximum (FWHM) and *θ* is the Bragg’s diffraction angle. 

Table 1 presents the values of these parameters calculated with Equations (1)–(3). As also shown in Figure 4, the crystallite size decreases to 13.5 nm for 2% Ag. It then increases to 19.6 nm for 5% Ag and decreases again for 10% Ag. The dislocation density of CMTS films is found to increase from 4.5 × 10^11^ lines/m^2^ for the undoped CMTS to 5.4 × 10^11^ lines/m^2^ for 2% Ag. Then, the dislocation density decreases to the lowest value for 5% Ag. This behavior correlates well with the improvement in crystallinity for Ag-doped CMTS obtained with 5% Ag. The strain caused by the lattice stress in the films shows a low value of 1.6 × 10^−3^ % for the sample obtained with 5% Ag. Therefore, we can conclude that 5% Ag added in the CMTS spray solution has a beneficial influence on the structural properties of CMTS.

#### 3.1.2. Raman Spectroscopy Analysis

**Figure 5:**
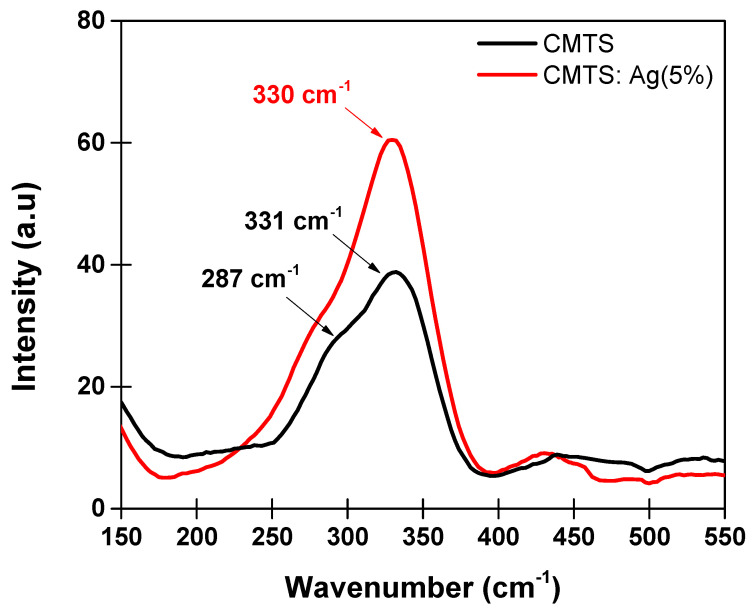
Raman spectra of undoped and Ag (5%)–doped CMTS thin films.

Figure 5 presents the Raman spectra of the undoped and Ag (5%)-doped CMTS thin films. It is clearly seen that the peaks are located at 331 cm^−1^ and 330 cm^−1^ for the CMTS and Ag (5%)-doped CMTS, respectively. This peak is generally related to the CMTS phase as reported in the literature [25], which is due to the anions vibration mode of CMTS Kesterite phase. Additionally, a shoulder peak located at 287 cm^−1^ for the CMTS spectrum can be seen, which also refers to the CMTS phase as found by Hammoud et al. [9,25]. Furthermore, no additional peaks are detected, which indicates the absence of secondary phases and impurities in both doped and undoped CMTS. This finding is in agreement with the XRD results. 

#### 3.1.3. Morphological and Compositional Analysis

**Figure 6:**
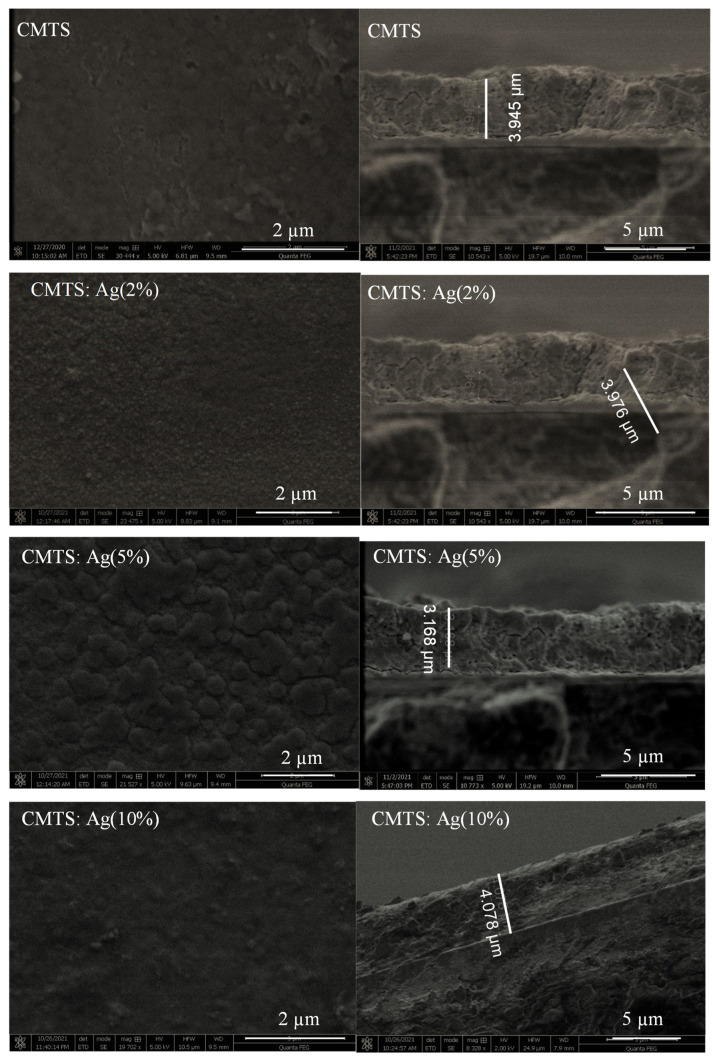
SEM micrographs of top view (**left column**) and cross-section (**right column**) of the undoped and Ag–doped CMTS films.

Figure 6 presents the SEM images of the undoped and Ag-doped CMTS thin films with several Ag/(Ag + Cu) ratios (i.e., 2%, 5%, and 10%). SEM images show a relatively smooth surface with a texture that could be caused by Ag incorporation [24]. For the undoped CMTS thin film, we observed a smooth and uniform surface, which contains irregular nearly spherical-shaped particles showing agglomerations in certain areas. When the Ag was 2%, the appearance of the CMTS surface slightly changed, showing small spheres that resemble small bubbles. When the Ag content increased to 5%, the size of these spheres also increased. The aspect of the surface shows homogeneous features without any voids or cracks, which also indicates the good crystallization of this material. Moreover, Gunavathy et al. [26] showed that the spherical grains improve the optical and electrical properties of CZTS. Large, dense grains have fewer boundaries, which can reduce the carrier recombination and improve the efficiency of solar cells [27,28]. Finally, for the 10% Ag CMTS, the surface of the film was uniform and smooth with small grains similar to that of the CMTS thin film grown with Ag = 2%. The same observations were reported by Nguyen et al. [18] for the Ag-doped CZTS thin films.

The right column of Figure 6 shows the cross-section of the films. The thickness of the films was estimated as a function of Ag doping. The thickness of the film increases, then decreases and then increases again for the Ag amount of 2%, 5% and 10%, respectively. The thickness values were estimated as 3.945 µm for CMTS and 3.976 µm, 3.168 µm and 4.078 µm for 2%, 5% and 10% Ag CMTS, respectively. Therefore, the thinnest layer was observed for 5% Ag, which can be explained by a more ordered and better structured crystallization of the CMTS thin films for this doping level [29]. These results correlate well with the structural analysis presented above in Figure 4 and Table 1. 

**Figure 7:**
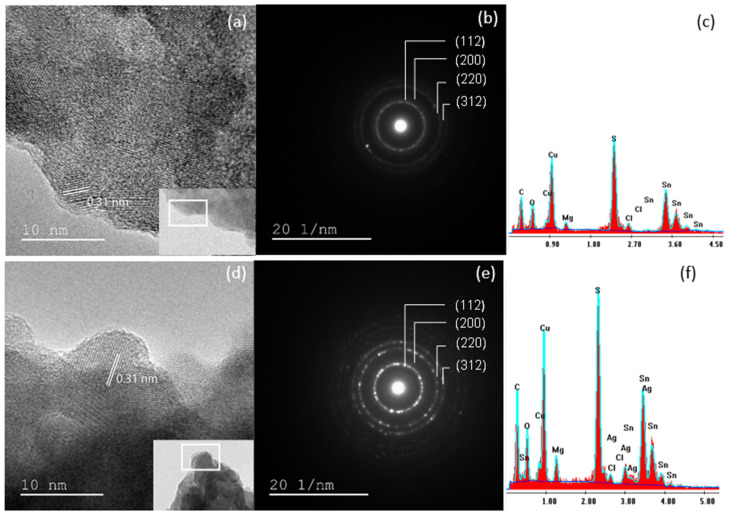
TEM images, HR-TEM images, SAED rings and the EDAX spectra of undoped (**a**–**c**) and 5% Ag CMTS particles (**d**–**f**).

In order to better understand the structure of the films, transmission electron microscopy, high-resolution transmission electron microscopy images and the associated selected area electron diffraction of the undoped CMTS (Figure 7a–c) and 5% Ag CMTS (Figure 7d–f) particles were performed. Figure 7a,b show the TEM and HR-TEM images of CMTS particles, where the reticular inter-planar (112) spacing is in the order of 0.31 nm, which is close to the reference file of this material (JCPDS00-026-0575). Hammoud et al. [9] obtained similar results for CMTS. Moreover, the TEM images in Figure 7a,d show an agglomeration of spherical particles for both the undoped and 5% Ag CMTS. In the inset of Figure 7a,d, the agglomeration is better observed and can be associated with the SEM images presented above. SAED images (Figure 7b,e) reveal the polycrystalline nature of CMTS particles, which is indicated by the presence of diffraction rings corresponding to the (112), (200), (220), and (312) reticular planes. These results are in good agreement with the DRX analysis. Figure 7c,f present the energy dispersive X-ray analysis (EDAX) results of the undoped and 5% Ag CMTS, which show the elemental composition of the CMTS and the presence of Ag in the doped CMTS sample. The presence of carbon and oxygen peaks in EDAX spectra is usually related to residues that cause detector contamination. Additionally, the presence of Cl is related to the use of metal chlorides as precursors (copper, magnesium and tin dichloride) in the spray solution, which results in residual chloride products that may not be completely evaporated. For 5% Ag CMTS, the elemental composition of the film is given by MgK = 8.3 at.%, SK = 41.9 at%, AgL = 5.1 at.%, CuK = 34.5 at.%, SnK = 10.3 at.%, which result in a (Ag/Ag + Cu) ratio of 0.12% in the Ag-doped CMTS film.

### 3.2. Optical Properties 

**Figure 8:**
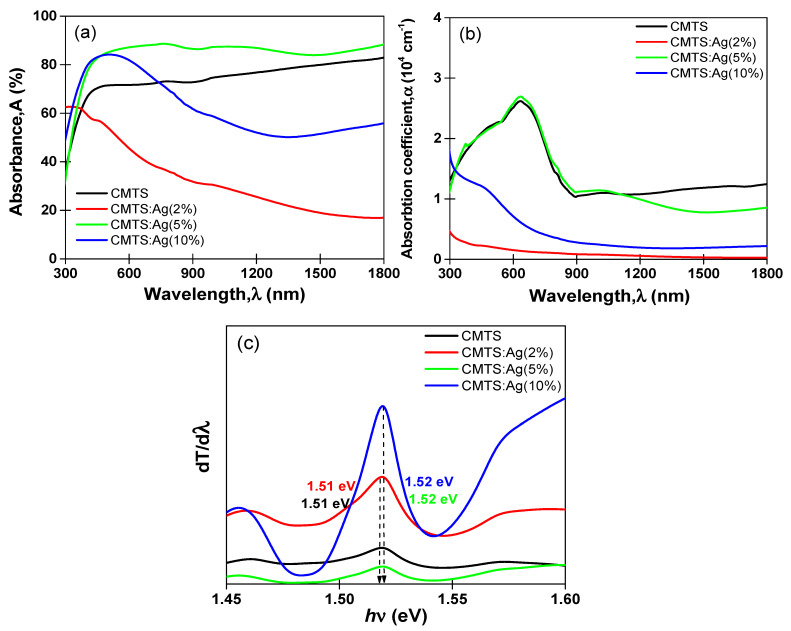
Absorbance (**a**,**b**) absorption coefficient, α, spectra and (**c**) the plot of *dT/dλ* versus *hν* for the undoped and Ag–doped CMTS films.

Figure 8a,b show the optical absorbance (A) and absorption coefficient (*α*) spectra. The undoped and 5% Ag CMTS films present two behaviors. First, in the UV range, the absorbance increases until it reaches its highest value of about 75% and 90% for the undoped and 5% Ag CMTS, respectively. Then, there is a stabilization tendency for the transition region from visible to IR. These results are in agreement with those obtained by our group on CMTS [9], which suggest that the exceptional absorbance of the CMTS films may make their use as an absorber layer in photovoltaic applications recommendable. In the case of Ag = 2%, a decrease from the maximum absorbance value in the range 300–500 nm from about 65% to low value in IR region. For 10% Ag CMTS, an increase until a high absorbance value more than 80% in the visible range and then decreases of about 50% in IR range are seen.

The absorption coefficient (*α*) was estimated using the following relation [23] based on transmittance (*T*), reflectance (*R*) and the thickness (*e*) obtained from the SEM cross-section images:

(4) $$\alpha =\frac{-1}{e}\;ln\;\left[\frac{T\left(\lambda \right)}{{\left(1-R\left(\lambda \right)\right)}^{2}}\right]$$

As presented in Figure 8b, the dependence of the wavelength on the absorption coefficient shows that all films exhibit a high absorption coefficient in the visible region in the order of (0.5–3.5)·10^4^ cm^−1^. The highest absorption coefficient of about 2.5 × 10^−4^ cm^−1^ was obtained for both undoped and 5% Ag CMTS. This value can affect the photovoltaic properties of CMTS films [30,31,32]. The band gap energy (*Eg*) using the differential transmittance (*T*) spectra of the films against the incident photon energy (*hv*) is illustrated in Figure 8c. One can notice that the undoped CMTS film has a band gap energy of 1.51 eV and the Ag-doped CMTS films obtained with 2%, 5% and 10% have *Eg* values of 1.51 eV, 1.52 eV and 1.52 eV, respectively. Nguyen et al. [24] studied the effect of Ag incorporation into the CZTS films grown by spray pyrolysis and observed a slight change in band gap energy after Ag incorporation (i.e., 1.48 eV and 1.47 eV). These results again demonstrate the good optical properties of the CMTS thin film and its seemly application in the solar cells.

### 3.3. Electrochemical Impedance Spectroscopy Measurements

The electrical properties of undoped and Ag (2%, 5% and 10%)-doped CMTS films were analyzed by electrochemical impedance spectroscopy. The impedance data can be plotted in two ways: (i) plotting the imaginary part (−*Z*″) versus real part (*Z*′), which is known as the Nyquist plot; and (ii) plotting the magnitude (|*Z*|) and/or phase angle (*Φ*) versus *log f*, which is known as the Bode plot [26]. Herein, we measured the impedance of CMTS films by applying 1V of alternative current (AC) and varying the frequency in the range of 20 Hz–2 MHz under dark and illumination [33] using Agilent E4980 impedance.

**Figure 9:**
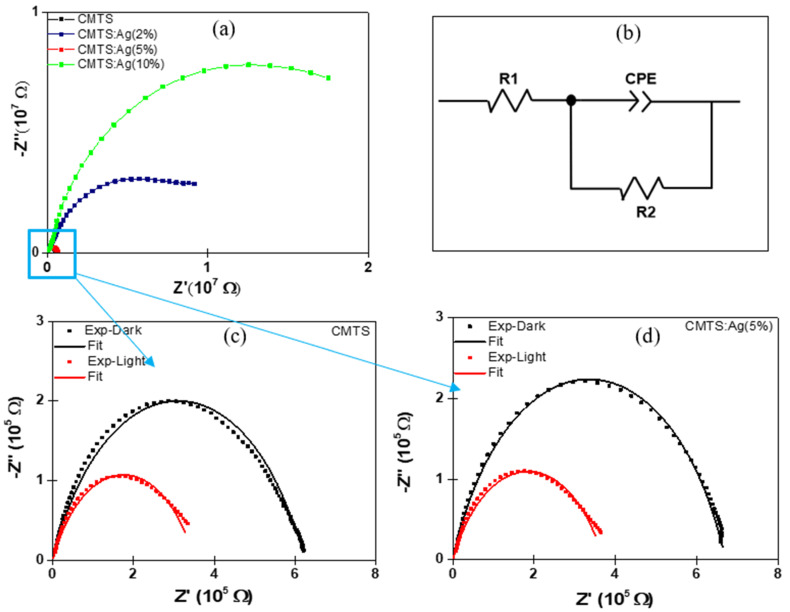
Nyquist plot of (**a**) undoped and Ag (2%, 5% and 10%)–doped CMTS films, (**b**) impedance equivalent circuit of CMTS and (**c**,**d**) Nyquist plot of undoped and 5% Ag CMTS films under dark and light.

Figure 9a presents the Nyquist plots (−*Z*″ vs. *Z*′) for all of the samples. For the Ag-doped CMTS with 2% and 10%, a large semicircle with a diameter in the order of 10^7^ Ω was observed, whereas for the undoped and 5% Ag CMTS, a small semicircle with a diameter of about 6 × 10^5^ Ω was observed, which indicates their good electrical properties. The semicircles obtained for the undoped and 5% Ag CMTS were modelled by the equivalent circuit presented in Figure 9b, which involves a series resistance of the electrode system (R1) in series with a charge transfer resistance (R2) parallel connected to a capacitance CPE where CPE is the constant phase element, defined by CPE-T and CPE-P, which are associated to the interface capacitor and an ideal capacitor [34].

The Nyquist plots of the undoped and 5% Ag CMTS under dark and illumination conditions are presented in Figure 9c,d. It is clearly shown that semicircles appear in both samples, indicating the Debye relaxation type [35]. Additionally, the diameter of the semicircle is significantly reduced for dark compared to illumination, which means the increase in the charge transport process under illumination [36]. These results are similar to previous reports on CMTS films [2].

The parameters of the equivalent circuit of CMTS films obtained by fitting the impedance data are summarized in Table 2. It is notable that the *R1* values of both samples are almost constant (10^−8^ Ω), indicating that *R1* is independent of illumination [37]. Moreover, the smallest radius is observed for both undoped and 5% Ag CMTS under illumination. Thus, the *R2* values are found to be dropped down from 6·10^5^ Ω in the dark to 3.5 × 10^5^ Ω under illumination, for both undoped and Ag-doped CMTS.

**Figure 10:**
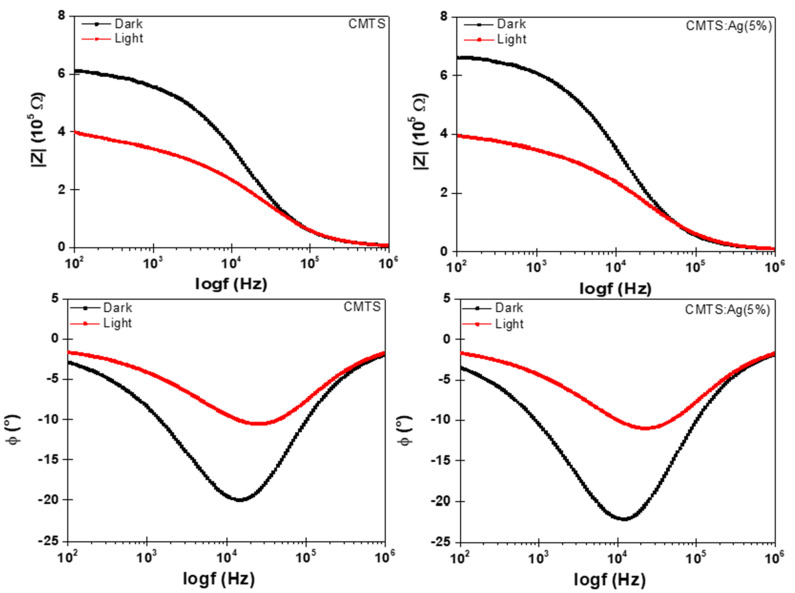
Bode impedance magnitude and phase angle plots of undoped and 5% Ag CMTS films under dark and illumination conditions.

Figure 10 presents the impedance magnitude (|*Z*|) versus semi-logarithmic scales of frequencies (log*f*). It can be seen that the impedance in the low-frequency region, i.e., from 10^2^ Hz to 10^3^ Hz, gives the total resistance value of the films [38]. Then, the impedance magnitude decreases and reaches the lowest value at high frequencies, i.e., in the region of 10^6^ Hz. These results show the increase in film conductivity [39].

Moreover, at high frequencies, there is no change observed in either the dark or under illumination, which confirms that the ohmic resistance (*R1*) is independent of high frequencies for both undoped and 5% Ag CMTS films. Similar results were previously found by Sharma et al. [2]. Furthermore, in Figure 10, the phase angle (*Φ*) of the undoped and 5% Ag CMTS films exhibits one peak at intermediate frequencies, i.e., 10^4^ Hz to 10^5^ Hz, which can be modeled as a constant phase element in parallel with resistance.

### 3.4. Antibacterial Study

In order to determine the antibacterial activity of CMTS and CMTS:Ag (5%) particles, the diameter of the inhibition zone around the discs was evaluated.

**Figure 11:**
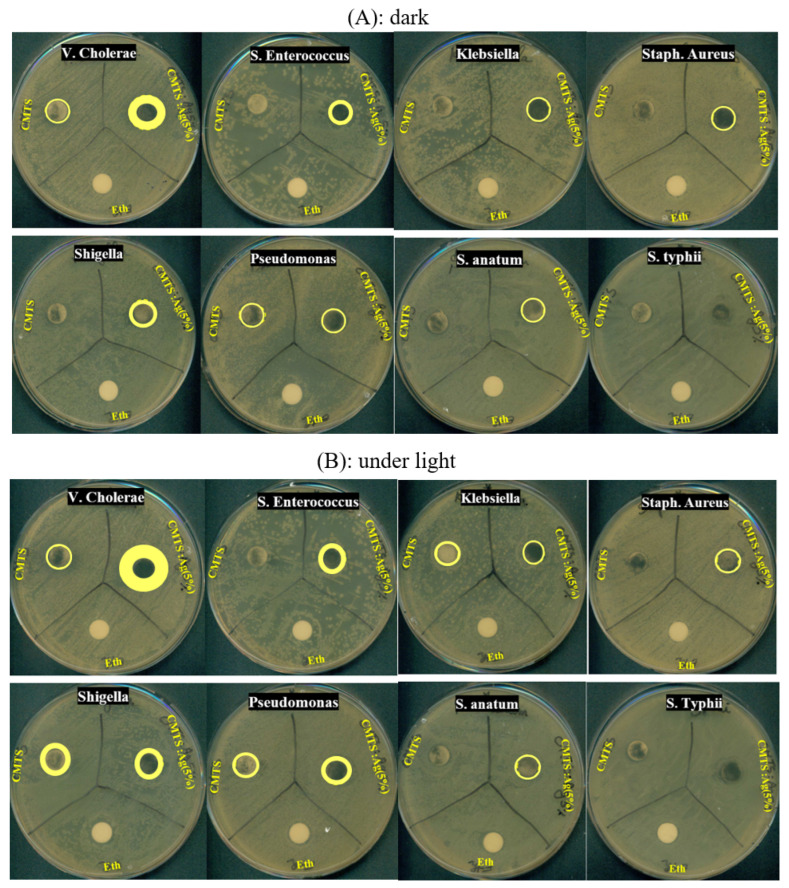
Photographs of the antibacterial test under dark (**A**) and light (**B**) conditions against various bacteria using the agar-well diffusion method, for CMTS, CMTS:Ag (5%) and negative control (Eth).

**Figure 12:**
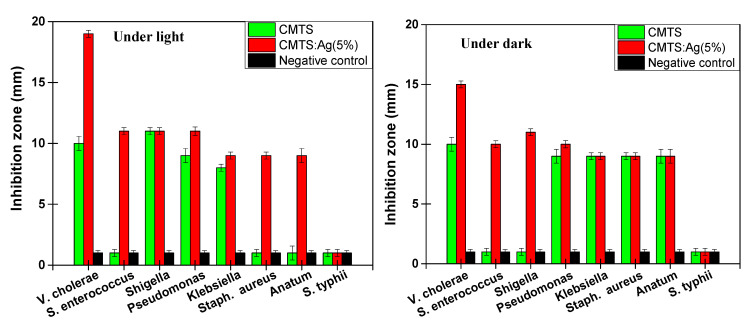
The variation in the inhibition diameter for each corresponding bacteria for the undoped (green), 5% Ag CMTS (red) and negative control (black) under dark (**left**) and light (**right**) conditions.

Figure 11A,B present the photographs of antibacterial tests for CMTS and 5% Ag CMTS particles and a negative control, ethanol, against eight bacterial species (i.e., *V. cholerae*, *S. enterococcus*, *Shigella*, *Pseudomonas*, *Klebsiella*, *Staph. aureus*, *S. anatum* and *S. typhii*). Figure 12 presents the variation in the inhibition diameter for each corresponding bacteria, under dark and light irradiation, respectively. Under dark conditions (Figure 11A), the CMTS particles exhibit a small inhibition zone for V. Cholerae and Pseudomonas bacteria, whereas the *S. enterococcus*, *Shigella*, *Klebsiella*, *Staph. aureus*, *S. anatum* and *S. typhii* do not have an inhibition zone, which means that CMTS particles with a concentration of 15 mg/mL are not able to reduce the growth of these types of bacteria. However, 5% Ag CMTS particles present a remarkable inhibition zone around wells, especially for V. Cholerae and Pseudomonas bacteria; these results indicating the impact of the Ag amount on bacterial growth. 

Figure 11B shows the antibacterial effect after 24 h of incubation under light irradiation, where a significant diameter of the inhibition zone of about 19 mm for a sample prepared with 5% Ag CMTS against V. Cholerae bacteria can be observed. These results indicate the bacteriostatic ability of our samples [19], while a small diameter for CMTS films (i.e., 10 mm) was observed. These results also mean a low resistance of this non-doped sample against *Shigella*, *Pseudomonas*, and *S. enterococcus*, with various inhabitation zones, as shown in Figure 12. In fact, it can be seen that under irradiation, the Ag-doped CMTS particles reveal a significant antibacterial activity for all the tested bacteria except for *S. typhii*, when compared to the negative control. It should be mentioned that this is the first report demonstrating the antibacterial activity of CMTS and Ag-doped CMTS nanoparticles synthesized by spray pyrolysis. Ali et al. [7] reported that under light, the CMTS-BTO heterojunction (where BTO is BaTO_3_) can be degraded by both pathogenic Gram-positive (*S. aureus*) and Gram-negative (*E. coli*) bacteria from contaminated waters due to the carriers generated from BTO and higher absorption of CMTS. Moreover, the same findings were previously observed by Kumar et al. [19] for the antibacterial activity of Cu_2_ZnSnS_4_ nanoparticles. Under light conditions, the CMTS generates more electron–hole pairs (e–h) than in dark conditions, which was shown in the previous section (i.e., electrochemical impedance spectroscopy measurements). 

To correlate these findings on antimicrobial activity, several mechanisms have been reported [7,11,19]. In fact, there are two types of bacteria based on their structure. Gram-positive bacteria have a thicker peptidoglycan layer compared to Gram-negative ones [40], which explains the high antibacterial effect of 5% Ag CMTS against V. cholerae (Gram negative). Additionally, it is known that Cu has a high antibacterial impact [41], as does the Mg metal [42]. Additionally, Ag nanoparticles have a noticeable resistance toward various types of bacteria, as reported by Jalal et al. [43]. Therefore, in other works, bacteria can be killed by the release of metal cations by dissolution from CMTS particles, such as Cu^2+^ and Mg^2+^, which can oxidize the bacteria wall, causing the rupture of this wall and the loss of bacterial cell components [18]. Furthermore, Jalal et al. [43] reported that Ag NPs can be attached and accumulated in the cell wall but can also penetrate via ion channels into the cells, which leads to mechanical destruction of the cell [43]. 

The TEM images show that the size of the particles is in the order of 14 nm for CMTS, which increases the surface area and leads to direct interactions with microorganisms [34]. Indeed, 5% Ag CMTS have a notable inactivation under light irradiation occurred against V. cholerae, enterococcus, Shigella and pseudomonas bacteria due to the absorption edge of CMTS, which leads to the photocatalytic reaction; the reduction and oxidation process can be initiated by e-h generation, which helps the production of hydroxyl groups and oxygen species that have a direct impact on the bacterial wall [7,11].

## 4. Conclusions

In summary, CMTS and Ag-doped CMTS films obtained using different Ag/(Ag + Cu) ratios of 2%, 5% and 10% were synthesized by the spray pyrolysis technique. The as-prepared films show a tetragonal kesterite crystal structure with (112) as a preferred orientation for both undoped and Ag-doped CMTS. The shift in the peak to lower angles for the Ag-doped samples confirms a well incorporation of Ag. The SEM and TEM observations have shown a morphology characterized by nanometer-sized spherical particles. The crystallinity was verified by HR-TEM, where 0.31 nm inter-planar spacing on (112) was measured. Furthermore, the SAED pattern confirmed the ideal crystalline nature of CMTS and Ag-doped CMTS nanoparticles. A high absorbance behavior was obtained for 5% Ag CMTS in visible and IR regions. The optical absorption spectrum shows a significant absorbing property with an optical band gap value of 1.51 eV and 1.52 eV, which is also suitable for solar cell applications. The electrochemical impedance survey shows a good electrical property under illumination for both undoped and 5% Ag CMTS, which indicates good photo-sensing properties of the films. CMTS and 5% Ag CMTS particles show good antibacterial potency against various types of bacteria.

## Figures and Tables

**Table 1:** Crystallite size (*D*), dislocation density (*δ*), strain (*ε*) calculated along the preferred orientation (112) of undoped and Ag-doped CMTS films.

Samples	2θ (°)	*D* (nm)	*δ* (10^11^ Lines/m^2^)	ε (10^−3^%)
**CMTS**	28.6	14.8	4.5	2.1
**Ag (2%)-CMTS**	28.55	13.5	5.4	2.4
**Ag (5%)-CMTS**	28.55	19.6	2.6	1.6
**Ag (10%)-CMTS**	28.55	13.9	5.1	2.2

**Table 2:** Parameters employed for impedance spectra fitting by using an equivalent circuit model.

Samples		R1 (10^−8^ Ω)	R2 (10^5^ Ω)	CPE-T (10^−9^ F)	CPE-P (F)
CMTS	Dark	1	6.1	4	0.75
	Light	1	3.5	4	0.70
CMTS:Ag (5%)	Dark	1	6.5	1	0.75
	Light	1	3.7	0.1	0.68

## Data Availability

The data presented in this study are available on request from the corresponding author.
